# Associations between Socioeconomic Status and Psychological Distress: An Analysis of Disaggregated Latinx Subgroups Using Data from the National Health Interview Survey

**DOI:** 10.3390/ijerph20064751

**Published:** 2023-03-08

**Authors:** Anna-Michelle Marie McSorley, Adrian Matias Bacong

**Affiliations:** 1 Center for Anti-racism, Social Justice, and Public Health, School of Global Public Health, New York University, New York, NY 10003, USA; 2 Center for the Study of Racism, Social Justice and Health, Fielding School of Public Health, University of California, Los Angeles, CA 90095, USA; 3 Stanford Center for Asian Health Research and Education, Division of Cardiovascular Medicine, Stanford University School of Medicine, Stanford, CA 94305, USA

**Keywords:** psychological distress, SES, Hispanics, Latinos, National Health Interview Survey

## Abstract

Differences in socioeconomic status (SES), including income, education, and employment, continue to be significant contributors to health disparities in the United States (US), including disparities in mental health outcomes. Despite the size and diversity of the Latinx population, there is a lack of literature describing differences in mental health outcomes, including psychological distress, for Latinx subgroups (e.g., Dominican, Puerto Rican, Cuban). Therefore, we used pooled data from the 2014–2018 National Health Interview Survey to examine variations in psychological distress among Latinx subgroups as compared to other Latinx subgroups and non-Latinx whites. Additionally, we conducted regression analyses and tested whether race/ethnicity modified the relationship between SES indicators and psychological distress. Findings indicate that individuals categorized as Dominican and Puerto Rican were among the Latinx subgroups with the highest levels of psychological distress when compared to other Latinx subgroups and non-Latinx whites. Additionally, results demonstrate that SES indicators, such as higher levels of income and education, were not necessarily significantly associated with lower levels of psychological distress for all Latinx subgroups when compared to non-Latinx whites. Our findings discourage the practice of making broad generalizations about psychological distress or its associations with SES indicators to all Latinx subgroups using results garnered from the aggregate Latinx category.

## 1. Introduction

In the United States (US), the Census Bureau estimates that 19% of the population identifies as Hispanic/Latino/a/x (henceforth referred to as Latinx) [1,2]. People of Latinx background are the second fastest-growing ethnic group in the US, and it is projected that the Latinx population will reach 111 million by 2060 [3]. Despite being racially or ethnically categorized as one large Latinx group, there is a great deal of diversity within this broad category. Those who identify as Mexican comprise 37.2 million Latinx, the largest subgroup, followed by 5.8 million Puerto Ricans, nearly 2.5 million Salvadorans, 2.4 million Cubans, and more than 2.3 million Dominicans [4]. This does not include the millions of people who identify as part of the 11 remaining largest Latinx subgroups in the US [4]. Despite the size and diversity of the Latinx population, there is a lack of health disparities literature that elucidates the differences in health outcomes, particularly mental health outcomes, for those nested within this larger demographic variable.

Broadly, the largest body of research on population-level Latinx mental health outcomes represents the results of the aggregate group [5,6,7]. Several studies have demonstrated that those within the aggregate Latinx group have a lower prevalence of depressive symptomology [8], psychiatric disorders [5], and non-specific psychological distress [9] when compared to non-Latinx whites in the US. Studies also indicate that those who identify as Latinx are overrepresented in under-resourced communities and are commonly exposed to social stressors, such as limited access to financial resources, employment, and educational opportunities, which are known to cause poor health outcomes [10]. At first glance, the presence of better mental health is at odds with the presence of socioeconomic disadvantage. However, these findings are consistent with the “Latinx Health Paradox”—an epidemiologic phenomenon in which lower mortality rates (and other poor health outcomes) are observed amongst those categorized as Latinx in the US, despite the high levels of exposure to social conditions known to increase mortality and produce poor health outcomes [6,10,11]. However, here again, the empirical evidence that composes the foundation of this paradox is largely reflective of the social exposures and health patterns observed among the aggregate Latinx group.

Given that the US Latinx population is composed of individuals with connections to 20 distinct Latin American countries, there are several reasons to question the generalization of observations from the aggregate, on mental health outcomes, social exposures, and their paradoxical associations, to this diverse group. First, as the largest Latinx subgroup in the US, observations made among Mexican/Mexican Americans have been driving the literature on the Latinx Health Paradox for decades [12]. As a result, Latinx health researchers have cautioned against the practice of ascribing these paradoxical observations to all Latinx subgroups [13]. Second, studies indicate that the paradox is most applicable to Mexican immigrants (and other recent migrants), with the initial health advantages eroding in subsequent US-born generations [12,13]. Third, evidence suggests that while health advantages are observed among Mexicans in the US for several health conditions (e.g., hypertension), despite socioeconomic disadvantages, this does not hold across all health outcomes (e.g., obesity), particularly when compared to other racial/ethnic groups in the US [14]. Lastly, while the current literature is scant, previous investigations have noted significant variations in mental health outcomes and their social correlates, including differences in the association between socioeconomic indicators and psychological distress across Latinx subgroups in the US [15]. This indicates that the practice of ethnic aggregation in the mental health literature likely masks the presence of mental health disparities, as well as the variations in social exposures that differentially shape these outcomes, for Latinx subgroups.

### 1.1. Mental Health and Latinx Subgroups in the US

Previous studies have pointed to significant differences in mental health outcomes across Latinx subgroups [16,17]. An investigation exploring subgroup-specific variations within the Latinx population, using data from the National Latino and Asian American Study, found that the odds of having a depressive or anxiety disorder were higher among those who identified as Puerto Rican than those who identified as Cuban or Mexican [5]. This is consistent with a number of studies that have found a greater prevalence of mental health disorders and disparities among Puerto Ricans when compared to other Latinx subgroups and non-Latinx whites in the US [16,17,18]. Studies have also identified a lower prevalence of depression among Mexicans when compared to Cubans and Puerto Ricans [19]. Expanding beyond the three largest Latinx subgroups in the US, a mixed-methods study among Central American youth, which included migrants from El Salvador, Honduras, and Guatemala, reported elevated levels of risk for psychological distress, post-traumatic stress disorder, depression, and anxiety among these subgroups [20]. Although the subgroup-specific literature remains limited, it points to a great deal of variability that could benefit from additional disaggregated analyses of mental health outcomes and the social factors that distinctly shape these outcomes among those who identify as Latinx in the US.

### 1.2. Sociodemographic Factors Influencing Mental Health among Latinx Subgroups

The extant literature has identified several social and demographic factors that distinctly influence mental health outcomes among Latinx subgroups [21,22], including nativity status [13], citizenship status [23], English proficiency [24], age [25], sex [26], and marital status [24,25]. A study of Cuban immigrants in South Florida found that more recent Cuban migrants were more likely to report worse levels of anxiety, self-esteem and depression than those who were well-established in the US [24]. These findings were linked to the presence of higher levels of English proficiency and social support amongst Cuban migrants who had resided in the US for a longer period as compared to new arrivals [24]. A recent study examining the influence of immigration stress and acculturation stress on mental health outcomes for six distinct Latinx subgroups (i.e., Mexican, Puerto Ricans, Cuban, South Americans, Central American, and Dominican), revealed that individuals who identified as Mexican had higher levels of stress related to issues of immigration and acculturation than individuals who identified as Cubans or Dominicans; however, these higher levels of stress were not significantly predictive of depression [26]. In this same study, age and gender emerged as significant correlates of depression among the six Latinx subgroups, with older adults and women reporting significantly higher levels of depression (as compared to younger adults and men, respectively). Similarly, a 2020 article on mental health among older Latinx adults presented evidence that indicated social risk factors for mental illness appeared to increase with age for Mexican, Puerto Rican, and Cuban subgroups in the US [25].

There are several additional relevant social distinctions to note across Latinx subgroups that impact mental health. For instance, among individuals who identify as Mexican, about a third are born outside of the US, and about 51% of Mexican immigrants do not have documentation to remain in the US [25]. As a result, a significant proportion of individuals from the Mexican community are overrepresented in precarious jobs (largely concentrated in the Western and Southwestern regions of the US), have lower education levels, and have limited access to supportive services that promote health and well-being in the US [21,27,28]. Conversely, Puerto Ricans are US citizens and do not experience barriers to accessing supportive services related to legal documentation status [29]. Most of the Puerto Ricans who reside in the 50 states were born in the US and benefit from high levels of English proficiency. However, Puerto Ricans continue to be socially and economically disadvantaged [25,29]. Indeed, substantial Puerto Rican enclaves are located in areas of the US traditionally characterized by extreme poverty and unemployment among minorities across the Northeastern regions of the US, with high concentrations residing in New York City and the South Bronx [30]. Distinctly, generations of Cubans have entered the US as refugees, which has granted many of them access to US social safety nets [24,25]. Cubans in the US have strong ties to the Southeastern region of the US, with large ethnic enclaves and strong social support structures in South Florida [24]. Literature also indicates that Cubans in the US have some of the highest levels of education and economic advantage when compared to other Latinx subgroups [21,25]. Given the variability in socioeconomic conditions and mental health outcomes across Latinx subgroups, additional exploration is warranted.

### 1.3. Socioeconomic Status as a Fundamental Cause of Mental Health Disparities

Decades of research point to a fundamental independent relationship between socioeconomic status (SES), which is inclusive of income, education, and employment factors, and health outcomes among racial and ethnic minorities in the US [31,32,33]. There is strong evidence and sound theoretical rationale, presented by Link and Phelan within Fundamental Cause Theory (FCT), that social conditions, such as low SES, give rise to risk factors for disease [31,32]. As a result of the great evidence that links low SES to poor health outcomes [27,34], SES has been identified as a fundamental cause—whereby low SES will always produce risk factors that lead to disease. A fundamental cause is so influential in the disease process that even when other proximal risk factors are removed, the fundamental cause will still give rise to poor health [31,32]. Therefore, SES is a crucial factor to examine as it relates to all health outcomes in all populations in the US.

As aforementioned, the Latinx Health Paradox has led to the assumption that the association between SES and health is universally paradoxical among all Latinx subgroups [11,13]. However, this does not appear to be the case, particularly when examining psychological distress—a strong predictor of the population’s mental health status [35]. In 2011, a study examining the relationship between wealth and psychological distress found that lower wealth possession was predictive of higher levels of psychological distress among Mexican, Puerto Rican, and Cuban subgroups [15]. Another investigation in New York City, where more than a quarter of people identify as Latinx, including high concentrations of individuals of Dominican, Puerto Rican, and Ecuadorian origin, found that Latinx had significantly higher rates of non-specific psychological distress when compared to non-Latinx whites [36,37]. Additionally, this disparity in psychological distress could be attributed to the disproportionately large concentration of people who were classified as Latinx with low SES [37]. Moreover, a study examining the effects of lifetime poverty in a nationally representative sample of young adults identified that Latinx had significantly higher rates of depression and demonstrated that unemployment and poverty were significantly associated with symptoms of depression among Latinx [38]. However, results in this study were not available at a Latinx subgroup level [38]. Overall, there remains a dearth of literature on subgroup-specific variations in the relationship between SES and psychological distress among Latinx in the US.

### 1.4. Specific Aims

**Figure 1:**
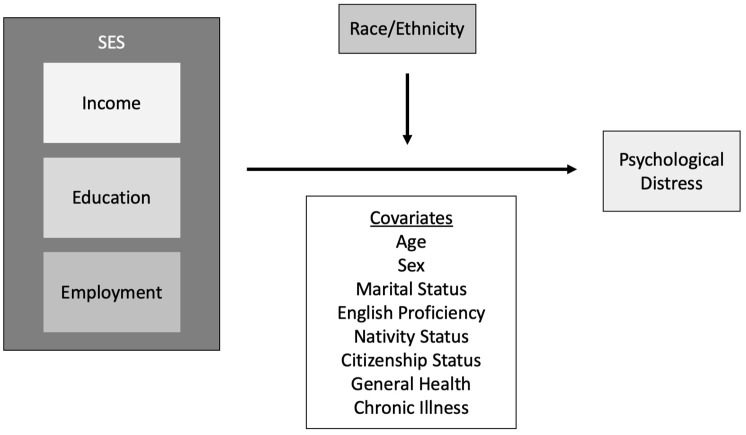
Conceptual Model for SES and Psychological Distress.

This study sought to address this gap in the literature by examining the relationship between SES and psychological distress across Latinx subgroups in the US through the execution of two aims. The first aim of this study was to describe variations in mental health, specifically psychological distress, among Latinx subgroups as compared to other Latinx subgroups and non-Latinx whites in the US. The second aim of this study was to determine whether race/ethnicity modified the relationship between (i) income and psychological distress, (ii) education and psychological distress, and (iii) employment and psychological distress (Figure 1).

## 2. Materials and Methodology

### 2.1. Study Design and Data Source

We used pooled public use data from the 2014–2018 National Health Interview Survey (NHIS) [39]. Two advantages of using the NHIS for this analysis are (1) the nationally representative Latinx sample with detailed subgroup categories due to oversampling [39] and (2) the consistency of items across administrations. Combining five years of NHIS data led to a sample of 463,974. However, our final analytical sample consisted of 71,064 individuals after restricting our analyses to Latinx and non-Latinx white adult participants with complete data. The datasets used for the analyses in this study, and all necessary materials and documentation for the NHIS, are available online from the National Center for Health Statistics (https://www.cdc.gov/nchs/nhis/index.htm, accessed on 2 October 2021) (Supplementary Materials). Due to the public nature of the data, this research is human subjects research exempt and not subject to review by the Institutional Review Board.

### 2.2. Measures

*Psychological Distress:* The Kessler 6 (K6) is a validated scale used to assess population-level, non-specific psychological distress [40]. The scale consists of six items that asked, “During the past 30 days, how often did you feel… (Item 1) so sad that nothing could cheer you up, (Item 2) nervous, (Item 3) restless or fidgety, (Item 4) hopeless, (Item 5) that everything was an effort, and (Item 6) worthless?” Each of these items were scored on a range from 0 (“none of the time”) to 4 (“all of the time”). Scores were summed to create a continuous measure of psychological distress, ranging from 0–24. We subsequently coded the K6 into three categories: those with (0) no distress to slight distress (Scores ≤ 5), (1) moderate distress (Scores 6–12), and (2) severe distress (Scores ≥13), based on recommendations from the literature [41]. Population studies have determined that a score of 13 or more is indicative of serious mental illness [35]. Additionally, measure validation has also identified the K6 as an appropriate measure for capturing moderate levels of non-specific psychological distress [41].

*Socioeconomic Status (SES):* SES was measured using income, education, and employment. *Income* was measured by an item on the NHIS questionnaire that asked participants to report total earnings last year and was presented as a categorical variable of income ranges, generally increasing by $5000 increments. This item had 14 potential options. In the interest of creating a more parsimonious model, income was recoded into five categories: Less than $15,000 (referent category), $15,000 to $34,999, $35,000 to $74,999, $75,000+, and unknown (which included observations collected from respondents who refused to state their income or reported that they did not know).

*Education* was measured by a single item that asked respondents to report their highest level of education by selecting from 23 potential categorical options. Education was recoded as a four-category variable: less than high school (referent category), high school graduate/GED or equivalent, some college/no degree, and college degree or advanced degree. Observations were dropped for respondents who refused to answer, selected the don’t know option, or in cases where the highest level of education could not be ascertained from the data. This is consistent with the coding of a previous investigation examining racial and ethnic differences in psychological distress using the NHIS [9].

*Employment* was measured by an item that asked about employment status within the last week and was presented on the questionnaire with seven possible options, which were recoded as a dichotomous variable to include employed (referent category) and unemployed. The employed category included individuals who reported: working for pay at a job or business, with a job or business but not at work, or working but not for pay at a family-owned job or business. The unemployed category included individuals who were looking for work or not working at a job or business and not looking for work. Individuals who endorsed the remaining options of “don’t know” and “refused” were removed from the analytic sample.

*Race and Latinx Subgroups:* Race/ethnicity were coded into seven categories: (1) non-Latinx white (referent category), (2) Mexican/Mexican American, (3) Central/South American, (4) Puerto Rican, (5) Cuban/Cuban American, (6) Dominican, and (7) Other Latinx/Multiple Latinx (not including Spaniards). Those who identify as belonging to multiple Latinx subgroups or smaller subgroups were categorized as “other”. As the majority of Latinx respondents identified as white (89.0%), Latinx ethnic categories were not racially categorized. People of Spanish ancestry were excluded from the Latinx category, given that typical conceptualizations of Latinx do not include European immigrants from the country of Spain. Non-Latinx white was chosen as the reference group because this group is the most populous and socioeconomically privileged group in the US.

*Covariates:* We accounted for possible confounding by demographic, family structure, acculturation, and health characteristics. Demographic controls included age, ranging from 18–84 years and sex (0 = male, 1 = female). Marital status was used as a measure of family structure and social support and coded as: currently married, formerly married, cohabitating, or never married. Acculturation included citizenship status (1 = US-born, 2 = Naturalized citizen, and 3 = Non-citizen) and English language proficiency (1 = very well/well, 2 = not well, 3 = not at all). Measures of general health status and chronic illness were also included. General health status was dichotomized as (0) indicating bad general health status and (1) indicating good general health status. Additionally, five key chronic illnesses (diabetes, coronary heart disease, stroke, cancer, and chronic obstructive pulmonary disease) were coded as dummy variables. Finally, we include “survey year” to account for any extraneous temporal confounders.

### 2.3. Statistical Analysis

Univariate analyses were conducted to assess individual variable characteristics. Bivariate analyses were conducted to assess relationships between each SES covariate (specifically, income, education, and employment) and psychological distress (the dependent variable of interest across all models). Chi-squared tests of independence were conducted to assess if any two categorical variables were related. Ordinal logistic regression analyses were utilized to assess the relationship between a three-category variable for psychological distress and SES by race and ethnicity. We tested if the association of each SES indicator was moderated by race/ethnicity separately (i.e., interaction terms for income by race/ethnicity, education by race/ethnicity, and employment by race/ethnicity were tested in distinct models). In total, five models were assessed. Model 1 assessed the independent effects of income, education, and employment on psychological distress. Model 2 assessed the interaction effect of income by race/ethnicity on psychological distress. Model 3 examined the interaction effect of education by race/ethnicity on psychological distress. Model 4 explored the interaction effect of employment by race/ethnicity on psychological distress. Finally, Model 5 included the interactions of all SES indicators by race/ethnicity. All models accounted for potential sociodemographic confounders. Data cleaning and analysis were completed using Stata Version 17.0. We used the “svy” and “subpop” commands in Stata to apply survey weights to allow the sample to be representative of the US population and account for the complex design of NHIS. We also used the “margins” command to calculate the predictive probabilities and the “margins plot” command to graph the interactions.

## 3. Results

### 3.1. Sample Characteristics

Table 1 displays the weighted univariate characteristics for the sample and bivariate characteristics by Latinx subgroup. Overall, the sample was majority male, under 65 years old, currently married, with high English proficiency and educational attainment (i.e., college degree or above), and most participants were employed. There were distinct differences in these sociodemographic characteristics by Latinx subgroup. Most notably, English proficiency was lowest among Cubans, followed by Dominicans, then Central and South Americans. The proportion of naturalized citizens was highest among Dominicans, followed by Cubans/Cuban Americans, and then Central and South Americans. Mexicans/Mexican Americans and Central and South Americans had the highest proportion of non-citizens.

### 3.2. Associations between Income, Education, and Employment and Psychological Distress by Latinx Subgroup

Table 2 presents the weighted bivariate association of psychological distress by income, education, and employment for the full sample. Overall, 2.43% of the sample had severe psychological distress, while 17.20% of the sample had moderate psychological distress. There were statistically significant differences (*p* < 0.001) in the distribution of income, education, and employment. In general, lower levels of income, lower levels of educational attainment, and unemployment were associated with a higher prevalence of moderate or severe psychological distress.

Table 3 presents the bivariate associations of income, educational attainment, and employment on psychological distress, but within each race and ethnic group. Overall, severe psychological distress differed amongst racial and ethnic groups (*p* < 0.001). Moderate (20.80%) and severe psychological distress (5.73%) were highest among the Other Latinx/Multiple Latinx group and lowest overall among Cuban/Cuban American (Moderate: 11.80%; Severe: 2.44%). The association of lower income with greater psychological distress was statistically significant among all groups except for Cuban/Cuban American, Dominican, and Other Latinx/Multiple Latinx. However, the trend was in the expected direction for these groups. Greater education was significantly associated with lower psychological distress only amongst non-Latinx whites. While the association between education and psychological distress was not statistically significant among Latinx subgroups, the expected direction was seen amongst Puerto Rican, Cuban/Cuban American, Dominican, and Other Latinx/Multiple Latinx. In contrast, there was a relatively equal distribution of severe psychological distress by educational attainment among Mexican/Mexican Americans. Finally, unemployment was significantly associated with greater severity of psychological distress for all racial and ethnic groups (Table 3).

### 3.3. Race/Ethnicity as an Effect Modifier

**Figure 2:**
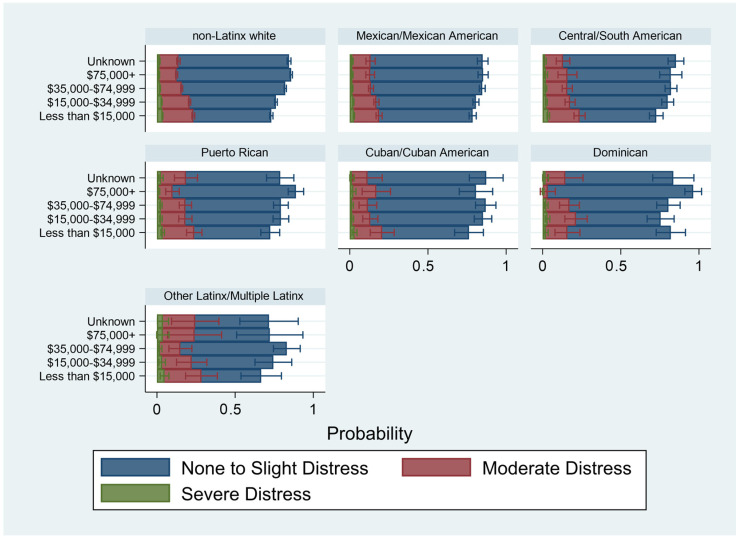
Predicted Probabilities of Level of Psychological Distress by Income and Racial/Ethnic Subgroup, 2014–2018 National Health Interview Survey (*n* = 71,064). Notes: Figure based on Table 4, Model 2. Accounts for demographic, social, and health factors.

**Figure 3:**
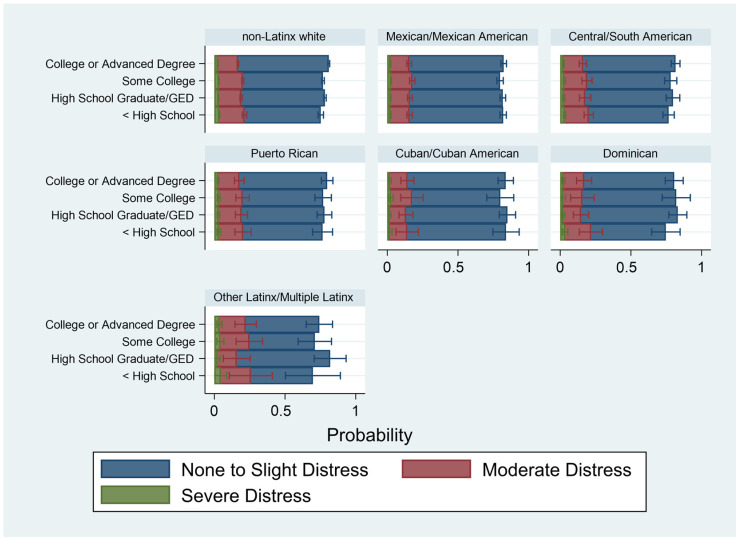
Predicted Probabilities of Level of Psychological Distress by Educational Attainment and Racial/Ethnic Subgroup, 2014–2018 National Health Interview Survey (*n* = 71,064). Notes: Figure based on Table 4, Model 3. Accounts for demographic, social, and health factors.

**Figure 4:**
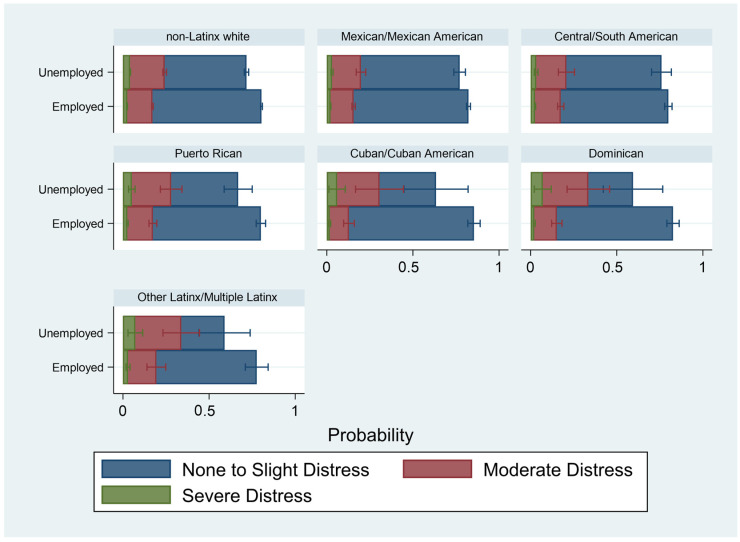
Predicted Probabilities of Level of Psychological Distress by Employment Status and Racial/Ethnic Subgroup, 2014–2018 National Health Interview Survey (*n* = 71,064). Notes: Figure based on Table 4, Model 4. Accounts for demographic, social, and health factors.

Table 4 presents the results of the weighted multivariable ordinal logistic regression of psychological distress on income, educational attainment, employment, and race/ethnicity. Model 1 examines the association of these key factors, accounting for demographic, social and health conditions. Compared to non-Latinx whites, only Mexican/Mexican Americans and Cuban/Cuban Americans had lower odds of worse psychological distress. All other groups had similar odds of worse psychological distress compared to non-Latinx whites. In general, the socioeconomic factors followed the expected trends. Greater income and having at least a college education were associated with lower odds of worse psychological distress. Being unemployed was associated with higher odds of worse psychological distress. These results were robust, considering demographic factors that could confound the association between indicators of SES and psychological distress. Figure 2, Figure 3 and Figure 4 examine the separate associations of income, educational attainment, and employment status on psychological distress, moderated by race and ethnicity (based on Table 4, Models 2–4). For ease of interpretation, these figures are presented as predicted probabilities.

In Figure 2, which examines the association of income with psychological distress, moderated by race/ethnicity, higher levels of income remain associated with higher predicted probabilities of “None to Slight” psychological distress for all race and ethnic groups. Similarly, lower levels of income were associated with higher predicted probabilities of moderate or severe distress. However, these patterns were not consistent by race and ethnic group. For example, predicted probabilities of moderate or severe psychological distress were distinctly high among Other Latinx/Multiple Latinx in the highest income category.

For education (Figure 3), higher levels of educational attainment were associated with higher predicted probabilities of “None to slight” psychological distress for all racial and ethnic groups. Lower levels of educational attainment were associated with higher predicted probabilities of moderate distress, but less so for severe distress. Predicted probabilities of severe distress were negligible across each race and ethnic group. It is worth noting, however, that the predicted probabilities of moderate distress were consistent across educational categories for Mexican/Mexican American, Central or South American, and Puerto Rican groups. In contrast, there was variance in the predicted probabilities of moderate distress among Cuban/Cuban American and Dominican groups. However, these differences were not statistically significant, given the overlapping 95% confidence intervals.

Finally, being employed (Figure 4) was associated with higher predicted probabilities of “None to Slight” psychological distress. However, some race and ethnic groups had little distinction in the predicted probabilities of the level of distress within certain employment categories. For example, predicted probabilities of “None to Slight” and moderate distress were not statistically different for Dominican and Other Latinx/Multiple Latinx groups.

## 4. Discussion

This study identified significant differences in psychological distress across all Latinx subgroups when compared to non-Latinx whites. However, there were some notable differences for specific subgroups. Individuals who identified as Dominican and Puerto Rican had some of the highest levels of severe psychological distress across Latinx subgroups and when compared to non-Latinx whites. For Puerto Ricans, these results are consistent with previous studies indicating that this Latinx subgroup experiences a disproportionate burden of psychological distress when compared to other Latinx and non-Latinx whites [15,42]. These elevated levels of psychological distress are likely linked to a host of contextual factors [23,43]. For instance, the dire economic circumstances [31] and severe natural disasters (e.g., Hurricanes Maria and Irma in 2017) that have contributed to mass migration from the island [44,45] have also had serious implications for psychological well-being and have been attributed to elevated levels of post-traumatic stress among Puerto Ricans [46]. As migration to the continental US continues in the coming years, these patterns of psychological distress may indeed worsen for the Puerto Rican population [45,46].

Distinct from the Puerto Rican subgroup, many individuals in the Dominican subgroup are Latinx immigrants who likely experience unfair treatment due to documentation status and experience a lack of access to social benefits—all of which have previously been documented as stressors that elevate psychological distress [23]. Importantly, the elevated levels of severe psychological distress observed among Dominicans highlight an alarming result that requires additional attention, particularly given this group’s ranking as the fifth-largest Latinx subgroup in the US [4]. Conversely, Cuban/Cuban Americans, in this and previous investigations [26], were among the subgroups with the lowest levels of psychological distress when compared to other Latinx subgroups and non-Latinx whites. Historically, Cubans have entered the county as refugees who receive resettlement benefits, including medical assistance, employment preparation, and job placement [47]. These social benefits likely reduce many of the structural stressors that are known to contribute to elevated levels of psychological distress among Latinx immigrants [23]. While descriptive, these significant variations in psychological distress by subgroup point to the dangers of generalizing results from studies conducted with an aggregate Latinx group onto all Latinx subgroups.

When examining the relationship between SES indicators (income, education, and employment) and psychological distress, several associations observed were significant and in the expected direction. In general, being employed was associated with lower levels of psychological distress for those included in the study. Similarly, higher levels of income were generally associated with lower levels of psychological distress for most Latinx subgroups and non-Latinx whites. However, several associations were significantly modified by race/ethnicity, with some notable Latinx subgroup differences. Compared to non-Latinx whites, Mexican/Mexican Americans had higher levels of psychological distress at the highest income level. Additionally, Mexican/Mexican Americans with a college education also appeared to have higher levels of psychological distress when compared to non-Latinx whites. While unexpected, previous studies among racially minoritized groups have demonstrated that higher levels of income and education are not necessarily protective against poor mental health outcomes, which has been linked to the elevated levels of discrimination experienced by these marginalized groups [48]. More specifically, studies have shown that individuals who are racialized as Mexican may have worse health as compared to people who are racialized as non-Latinx white in the US when accounting for socioeconomic characteristics [49]. This is consistent with the literature on Minorities’ Diminished Returns theory, which argues that educational attainment and income show a weaker protective effect for individuals belonging to racial and ethnic minority groups in the US when compared to non-Latinx whites [48,50,51].

With respect to employment, compared to non-Latinx whites, unemployed Dominican and Cuban subgroups had significantly higher levels of psychological distress. This is consistent with previous investigations looking at the effects of unemployment on mental health in the general population of the US [52]. However, as anticipated, these findings contradict the predominant literature in the Latinx health space, which indicates that Latinx have better health outcomes in the presence of worse socioeconomic standing [11]. Unfortunately, due to the limited nature of studies that examine mental health and SES among Latinx subgroups, particularly among individuals who identify as Dominican, we cannot point to previous literature that identifies similar trends. Moreover, we should note that although there are trends toward worse psychological distress for members of the Dominican and Cuban subgroups who are unemployed, the wide confidence intervals indicate that these findings should be interpreted with caution due to the limiting effects of small sample sizes. Lastly, among the remaining Latinx subgroups, where we observed no significant differences in the effects of income, education, and employment, we can surmise that our covariates may account for much of the difference that is observed in psychological distress for these subgroups when compared to non-Latinx whites.

### Strengths, Limitations, and Future Directions

As with any empirical investigation, there are important strengths and limitations to note. This study employs a cross-sectional design, and, as a result, causal conclusions cannot be drawn. Additionally, while an important strength of this study is its focus on heterogeneity, the groups presented in this piece were limited by existing NHIS categories and sample sizes. For example, Central and South American samples were combined by the NHIS data files but should be honored as distinct groups whenever possible. Although the subgroup categorizations were limiting, they do successfully highlight the diversity and variability that exists within the Latinx population and allowed us to provide new evidence about psychological distress among underexplored subgroups. For instance, to our knowledge, this is one of the first studies to describe patterns of psychological distress for Dominicans in the US.

Similarly, this is one of the few studies to demonstrate significant variations in psychological distress and its associations with SES indicators among Cubans, Puerto Ricans, and Dominicans. These are Latinx subgroups that are frequently aggregated into a Caribbean Latinx category, usually because of small sample sizes. Our findings highlight the need for oversamples within population health surveys to explore these and other health trends for the distinct Caribbean populations that are often lost in the general Latinx health literature [35]. Furthermore, the replication of these analyses in regions with high concentrations of individuals belonging to these subgroups (e.g., Northeastern US for Puerto Rican and Dominican subgroups or Southeastern US for Cuban subgroups) would be highly beneficial. As this investigation reveals, studies that continue to make broad claims and assumptions about the Latinx population in the US may be missing important differences within this group that contribute to our (mis-)understanding of population mental health outcomes and potential correlates, in this case, SES indicators.

## 5. Conclusions

This piece contributes to a growing body of literature within the field of Latinx health disparities that highlights the epidemiologic heterogeneity within the Latinx ethnic category, particularly as it pertains to psychological distress. This paper also contributes to the body of literature that confirms the presence of significant associations between SES (including income, education, and employment) and mental health outcomes among Latinx subgroups as compared to non-Latinx whites. Moreover, our findings indicate that the consequences of these social exposures are experienced differentially by race/ethnicity and do not support the generalization of the Latinx Health Paradox to all the Latinx subgroups examined. This is a significant contribution to the Latinx mental health literature, especially as the diversity within the Latinx community continues to increase in the US. Our findings suggest that specific subgroups within the Latinx community, including Puerto Ricans and Dominicans, require increased attention within the mental health space. This has meaningful implications for practice, as the members of these communities have distinct needs. For example, while Dominican immigrants may require support navigating stressors related to documentation status, Puerto Rican migrants may need unique levels of support surrounding post-traumatic stress resulting from consistent exposures to natural disasters. Understanding these intricacies has the potential to improve mental health among these subgroups.

Additionally, this descriptive piece provides baseline evidence needed by public health practitioners and researchers seeking to address mental health disparities within Latinx populations. Data and descriptive characteristics are essential components of community needs assessments, applications for grant funding, and community program development. However, Latinx subgroups have been lost for far too long within the aggregate Latinx group, which compromises the capacity of the public health community to address subgroups’ specific needs. Future investigations and public health interventions should continue to work towards honoring the heterogeneity that exists within the larger Latinx category.

## Figures and Tables

**Table 1:** Weighted Sample Characteristics in Percentages *(N =* 71,064).

Characteristics (%)		Non-Latinx White	Mexican/ Mexican American	Central/ South American	Puerto Rican	Cuban/ Cuban American	Dominican	Other/ Multiple Latinx	Full Sample
Sex									
	Male	50.96	54.38	53.53	49.44	58.73	42.86	48.39	51.36
	Female	49.04	45.62	46.47	50.56	41.27	57.14	51.61	48.64
Age									
	Under 65	90.83	96.87	95.62	96.88	92.42	97.11	95.98	91.76
	Over 65	9.17	3.13	4.38	3.12	7.58	2.89	4.02	8.24
Marital Status									
	Currently Married	48.51	46.57	47.04	36.99	48.28	40.63	29.89	47.93
	Formerly Married	19.3	15.45	16.27	18.23	17.89	22.26	18.07	18.80
	Co-Habitating	7.63	10.10	7.86	10.59	11.34	9.02	11.63	7.99
	Never Married	24.57	27.88	28.82	34.19	22.49	28.09	40.41	25.28
English Proficiency									
	Very well/Well	99.71	79.19	74.04	93.83	68.25	69.93	98.23	96.33
	Not well	0.24	14.53	19.38	5.02	18.32	22.04	1.36	2.63
	Not at all	<0.01	6.28	6.58	1.15	13.43	8.03	0.41	1.05
Nativity Status									
	US Born	94.95	52.72	19.39	63.62	31.45	22.51	82.88	86.83
	Foreign Born	5.05	47.28	80.61	36.38	68.55	77.49	17.12	13.17
Citizenship Status									
	US-Born	94.95	52.72	19.39	63.62	31.45	22.51	82.88	86.83
	Naturalized Citizen	3.57	16.06	37.58	35.23	39.87	50.56	9.81	7.03
	Non-Citizen	1.49	31.22	43.03	1.15	28.68	26.93	7.31	6.14
Income									
	Less than $15,000	16.45	21.49	21.37	19.32	14.56	21.75	20.69	17.19
	$15,000 to $34,999	22.52	36.31	33.80	29.93	34.46	32.78	28.37	24.56
	$35,000 to $74,999	33.32	25.71	25.34	29.59	27.31	24.19	30.29	32.13
	$75,000+	19.96	7.58	9.06	10.67	12.39	8.40	10.83	18.05
	Unknown	7.75	8.92	10.43	10.49	11.29	12.88	9.83	8.07
Education									
	Less than HS	4.18	28.99	22.20	13.74	10.97	19.63	8.24	7.55
	HS Graduate/GED	19.94	27.09	21.59	25.17	27.94	25.82	21.27	20.90
	Some College	19.48	18.48	18.44	19.89	13.01	19.17	24.93	19.33
	College/Advanced Degree	56.39	25.44	37.77	41.20	48.07	35.38	45.57	52.21
Employment									
	Employed	89.04	90.10	89.59	87.97	92.75	91.50	85.70	89.17
	Unemployed	10.96	9.90	10.41	12.03	7.25	8.50	14.30	10.83
Psychological Distress									
	Normal	80.26	81.65	80.77	77.10	85.75	79.73	73.47	80.37
	Moderate	17.40	15.71	16.35	19.72	11.80	16.17	20.80	17.20
	Severe	2.35	2.64	2.88	3.18	2.44	4.10	5.73	2.43
General Health									
	Good, Very Good, Excellent	94.39	90.83	93.04	91.10	93.98	90.88	94.13	93.87
	Fair, Poor	5.66	9.17	6.96	8.90	6.02	9.12	5.87	6.13
Chronic Illness									
	Diabetes (Yes)	5.08	6.64	4.31	5.81	5.11	6.08	4.97	5.23
	Heart Disease (Yes)	2.36	1.24	1.13	1.72	2.42	2.43	1.57	2.20
	Stroke (Yes)	1.18	0.67	0.96	0.66	0.25	0.78	1.43	1.10
	COPD (Yes)	1.71	0.51	0.47	0.81	1.15	<0.01	<0.01	1.51
	Cancer (Yes)	7.65	2.37	2.36	3.70	2.94	1.31	2.54	6.79
Sample Size		58,765	7601	2235	1183	580	399	301	71,064

Notes: COPD = Chronic Obstructive Pulmonary Disease; HS = High School; GED = General Educational Development Test.

**Table 2:** Psychological Distress by Income, Education and Employment for Weighted Full Sample.

		Full Sample (*N* = 71,064)					
		Normal		Moderate		Severe	
Variables		*n*	%	*n*	%	*n*	%
Psychological Distress		57,164	80.37	12,125	17.20	1775	2.43
Income ***							
	Less than $15,000	5930	15.55	2878	22.64	620	33.02
	$15,000 to $34,999	3485	23.30	3617	28.91	628	35.30
	$35,000 to $74,999	3152	33.06	3516	29.45	340	20.38
	$75,000+	10,851	19.66	1412	12.46	78	4.43
	Unknown	4295	8.43	702	6.55	109	6.87
Education ***							
	Less than HS	4495	7.28	1065	8.04	244	12.97
	HS Graduate/GED	12,211	20.62	2676	21.22	490	27.91
	Some College	10,678	18.47	2737	22.43	461	25.84
	College Degree	29,780	53.62	5647	48.31	580	33.27
Employment ***							
	Employed	51,501	90.34	10,444	86.00	1294	73.16
	Unemployed	5663	9.66	1681	14.00	481	26.84

Notes: *** Chi-squared test resulted in *p* < 0.001. Normal Psychological Distress indicates those with a K6 score < 5; Moderate Psychological Distress indicates those with a K6 score between 5 and 12; Severe Psychological Distress indicates those with a K6 score of 13 and above.

**Table 3:** Psychological Distress by Income, Education, and Employment among Latinx Subgroups and non-Latinx whites.

		Non-Latinx White			Mexican/Mexican American		
		*n* = 47,256	*n* = 10,106	*n* = 1403	*n* = 6167	*n* = 1216	*n* = 218
Variables		Normal	Moderate	Severe	Normal	Moderate	Severe
Psychological Distress **		80.26	17.40	2.35	81.65	15.71	2.64
Income							
	Less than $15,000	72.08	23.18	4.74	76.40	19.53	4.07
	$15,000 to $34,999	75.05	21.38	3.56	80.62	16.46	2.92
	$35,000 to $74,999	82.47	16.03	1.50	85.34	12.87	1.80
	$75,000+	87.62	11.83	0.56	86.61	12.38	1.02
	Unknown	84.31	13.72	1.97	83.59	14.47	1.94
		*p* < 0.001			*p* < 0.001		
Education							
	Less than HS	73.98	20.83	5.19	82.23	15.08	2.90
	HS Graduate/GED	78.66	18.06	3.28	82.26	12.87	2.87
	Some College	76.51	20.25	3.24	78.56	18.55	2.90
	College Degree	82.58	15.92	1.49	82.56	15.25	2.18
		*p* < 0.001			*p* = 0.118		
Employment							
	Employed	81.30	16.80	1.90	82.39	15.26	2.34
	Unemployed	71.77	22.26	5.96	74.87	19.74	5.40
		*p* < 0.001			*p* < 0.001		
Sample Size		58,765			7601		
		**Central/South American**			**Puerto Rican**		
		***n*** ** = 1795**	***n*** ** = 375**	***n*** ** = 65**	***n*** ** = 908**	***n*** ** = 236**	***n*** ** = 39**
**Variables**		**Normal**	**Moderate**	**Severe**	**Normal**	**Moderate**	**Severe**
Psychological Distress **		80.77	16.35	2.88	77.10	19.72	3.18
Income							
	Less than $15,000	72.86	22.36	4.79	70.66	23.42	5.92
	$15,000 to $34,999	81.09	15.87	3.04	76.17	19.67	4.17
	$35,000 to $74,999	84.19	14.40	1.41	78.00	19.58	2.41
	$75,000+	84.85	13.72	1.43	90.26	8.74	0.00
	Unknown	84.14	12.60	3.26	75.73	23.60	0.67
		*p* = 0.007			*p* = 0.175		
Education							
	Less than HS	77.44	18.76	3.80	73.71	19.65	6.64
	HS Graduate/GED	81.05	15.54	3.41	76.66	19.02	4.32
	Some College	78.79	16.91	4.30	76.33	20.71	2.96
	College Degree	83.54	15.12	1.33	78.88	19.69	1.43
		*p* = 0.081			*p* = 0.175		
Employment							
	Employed	81.39	16.08	2.52	79.21	18.40	2.40
	Unemployed	75.46	18.62	5.92	61.74	29.38	8.88
		*p* = 0.008			*p* < 0.001		
Sample Size		2235			1183		
		**Cuban/Cuban American**			**Dominican**		
		***n*** ** = 495**	***n*** ** = 71**	***n*** ** = 14**	***n*** ** = 316**	***n*** ** = 65**	***n*** ** = 18**
**Variables**		**Normal**	**Moderate**	**Severe**	**Normal**	**Moderate**	**Severe**
Psychological Distress **		85.75	11.80	2.44	79.73	16.17	4.10
Income							
	Less than $15,000	77.39	18.58	4.03	80.79	15.38	3.84
	$15,000 to $34,999	86.52	10.71	2.77	72.64	21.07	6.29
	$35,000 to $74,999	88.72	10.73	0.55	80.14	17.27	2.59
	$75,000+	84.29	13.89	1.81	97.06	2.94	0.00
	Unknown	88.63	6.65	4.71	83.94	11.60	4.46
		*p* = 0.506			*p* = 0.345		
Education							
	Less than HS	85.76	8.36	5.88	72.64	21.41	5.95
	HS Graduate/GED	86.50	11.40	2.10	82.61	13.53	3.86
	Some College	81.04	15.76	3.20	81.80	13.67	4.52
	College Degree	86.60	11.75	1.66	80.44	16.54	3.01
		*p* = 0.506			*p* = 0.770		
Employment							
	Employed	87.34	10.79	1.87	82.20	14.53	3.27
	Unemployed	65.44	24.74	9.82	53.12	33.86	13.02
		*p* = 0.002			*p* = 0.001		
Sample Size		580			399		
		**Other Latinx/Multiple Latinx**					
		***n*** ** = 227**		***n*** ** = 56**		***n*** ** = 18**	
**Variables**		**Normal**		**Moderate**		**Severe**	
Psychological Distress **		73.47		20.8		5.73	
Income							
	Less than $15,000	63.58		30.21		6.21	
	$15,000 to $34,999	74.03		17.8		8.17	
	$35,000 to $74,999	81.24		15		3.76	
	$75,000+	74.5		22.9		2.6	
	Unknown	67.6		25.2		7.2	
		*p* = 0.546					
Education							
	Less than HS	63.2		30.11		6.68	
	HS Graduate/GED	83		12.58		4.42	
	Some College	70.27		24.6		5.13	
	College Degree	72.63		20.87		6.5	
		*p* = 0.660					
Employment							
	Employed	77.13		17.54		5.32	
	Unemployed	51.5		40.32		8.18	
		*p* = 0.009					
Sample Size		301					

Notes: Chi-squared tests of independence were conducted for all predictors within Latinx subgroups. ** Chi-squared test resulted in *p* < 0.001.

**Table 4:** Weighted Multivariate Ordinal Logistic Regression of Psychological Distress on Income, Education, Employment, and Race/Ethnicity.

		Model 1		Model 2		Model 3		Model 4		Model 5	
Variables		OR	95% CI	OR	95% CI	OR	95% CI	OR	95% CI	OR	95% CI
Female Sex (Male Ref)		1.30 ***	1.24–1.36	1.29 ***	1.23–1.35	1.44 ***	1.38–1.51	1.41 ***	1.34–1.47	1.30 ***	1.24–1.36
Less than 65 years old (65+ Ref)		2.61 ***	2.35–2.89	2.51 ***	2.26–2.79	2.24 ***	2.03–2.48	2.44 ***	2.20–2.70	2.60 ***	2.34–2.89
Marital Status (Married Ref)											
	Formerly Married	1.66 ***	1.56–1.77	1.68 ***	1.58–1.78	1.66 ***	1.56–1.77	1.71 ***	1.61–1.82	1.66 ***	1.56–1.77
	Co-Habitating	1.60 ***	1.48–1.73	1.61 ***	1.48–1.74	1.71 ***	1.58–1.86	1.75 ***	1.62–1.90	1.60 ***	1.48–1.73
	Never Married	1.61 ***	1.52–1.71	1.63 ***	1.53–1.72	1.82 ***	1.71–1.93	1.81 ***	1.71–1.92	1.61 ***	1.52–1.71
English Proficiency (Very well/well Ref)											
	Not well	0.99	0.84–1.16	1.02	0.86–1.20	1.08	0.92–1.28	1.11	0.94–1.30	1.03	0.87–1.22
	Not at all	0.90	0.71–1.16	0.93	0.73–1.20	1.00	0.78–1.29	1.04	0.81–1.33	0.97	0.75–1.25
Born Outside of the U.S. (U.S. born Ref)		0.87 *	0.77–0.99	0.88 *	0.78–1.00	0.92	0.81–1.05	0.90	0.80–1.02	0.91	0.80–1.03
Naturalized Citizen (U.S. born Citizen Ref)		1.03	0.89–1.18	1.01	0.88–1.16	0.96	0.84–1.11	0.97	0.84–1.11	1.00	0.87–1.16
Survey Year (2014 Ref)											
	2015	1.17 ***	1.08–1.27	1.17 ***	1.08–1.26	1.16 ***	1.07–1.25	1.15 ***	1.06–1.24	1.17 ***	1.08–1.27
	2016	1.15 ***	1.06–1.24	1.15 ***	1.06–1.24	1.08 *	1.00–1.17	1.08 +	0.99–1.16	1.15 ***	1.06–1.24
	2017	1.27 ***	1.17–1.36	1.26 ***	1.17–1.36	1.18 ***	1.09–1.27	1.17 ***	1.09–1.27	1.27 ***	1.18–1.36
	2018	1.41 ***	1.30–1.53	1.40 ***	1.29–1.52	1.29 ***	1.19–1.40	1.30 ***	1.20–1.41	1.41 ***	1.30–1.53
Health Conditions											
	Fair/Poor Health (Good Health Ref ⊥)	3.72 ***	3.43–4.04	3.83 ***	3.53–4.16	3.97 ***	3.66–4.31	3.94 ***	3.63–4.28	3.72 ***	3.43–4.04
	Diabetes (None Ref)	1.15 **	1.04–1.28	1.16 **	1.05–1.28	1.15 **	1.04–1.27	1.15 **	1.04–1.27	1.15 **	1.04–1.27
	Coronary Heart Disease (None Ref)	1.11	0.95–1.31	1.12	0.95–1.32	1.14	0.96–1.34	1.12	0.95–1.32	1.11	0.94–1.31
	Stroke (None Ref)	1.54 ***	1.27–1.87	1.56 ***	1.28–1.89	1.56 ***	1.28–1.90	1.57 ***	1.29–1.91	1.54 ***	1.26–1.87
	COPD (None Ref)	1.57 ***	1.34–1.85	1.59 ***	1.36–1.87	1.64 ***	1.39–1.92	1.65 ***	1.41–1.94	1.57 ***	1.33–1.84
	Cancer (None Ref)	1.11 *	1.00–1.22	1.11 *	1.01–1.23	1.09 +	0.99–1.20	1.07	0.97–1.17	1.11 *	1.01–1.22
Race and Ethnicity (non-Latinx white Ref)											
	Mexican/Mexican American	0.79 ***	0.72–0.87	0.73 ***	0.63–0.85	0.65 ***	0.53–0.79	0.88 **	0.80–0.96	0.64 ***	0.50–0.82
	Central/South American	0.95	0.82–1.10	1.02	0.81–1.30	0.92	0.70–1.21	1.03	0.89–1.20	1.10	0.78–1.55
	Puerto Rican	1.01	0.86–1.20	1.04	0.75–1.44	0.93	0.60–1.44	1.02	0.85–1.23	0.82	0.48–1.39
	Cuban/Cuban American	0.74 *	0.55–0.99	0.84	0.49–1.45	0.55	0.26–1.19	0.68 *	0.50–0.93	0.55	0.23–1.33
	Dominican	0.89	0.67–1.18	0.58	0.30–1.13	1.03	0.57–1.87	0.85	0.65–1.12	0.52	0.22–1.23
	Other Latinx/Multiple Latinx	1.27	0.89–1.81	1.39	0.75–2.58	1.36	0.49–3.76	1.20	0.79–1.82	1.10	0.31–3.87
Income (<$15,000 Ref)											
	$15,000–$34,999	0.91 **	0.85–0.97	0.85 ***	0.79–0.91					0.93 *	0.86–1.00
	$35,000–$74,999	0.66 ***	0.61–0.71	0.58 ***	0.54–0.62					0.65 ***	0.60–0.70
	$75,000+	0.50 ***	0.46–0.55	0.43 ***	0.39–0.47					0.49 ***	0.45–0.54
	Unknown	0.55 ***	0.49–0.61	0.48 ***	0.43–0.54					0.52 ***	0.47–0.59
Race and Ethnicity × Income											
	Mexican × $15,000–$34,999			1.03	0.85–1.25					0.99	0.81–1.20
	Mexican × $35,000–$74,999			1.12	0.90–1.40					1.04	0.83–1.31
	Mexican × $75,000+			1.44 *	1.06–1.97					1.31 +	0.95–1.79
	Mexican × Unknown			1.31 +	0.96–1.80					1.26	0.91–1.72
	Central/South × $15,000–$34,999			0.77	0.54–1.08					0.73+	0.52–1.02
	Central/South × $35,000–$74,999			0.97	0.67–1.40					0.97	0.67–1.40
	Central/South × $75,000+			1.32	0.75–2.33					1.36	0.75–2.47
	Central/South × Unknown			0.91	0.55–1.50					0.87	0.53–1.43
	Puerto Rican × $15,000–$34,999			0.79	0.50–1.27					0.83	0.52–1.33
	Puerto Rican × $35,000–$74,999			1.17	0.74–1.84					1.23	0.76–1.99
	Puerto Rican × $75,000			0.72	0.39–1.34					0.77	0.40–1.50
	Puerto Rican × Unknown			1.44	0.74–2.78					1.44	0.75–2.76
	Cuban × $15,000–$34,999			0.64	0.32–1.25					0.72	0.33–1.56
	Cuban × $35,000–$74,999			0.80	0.37–1.76					0.97	0.38–2.50
	Cuban × $75,000+			1.75	0.71–4.33					2.16	0.75–6.18
	Cuban × Unknown			0.93	0.30–2.86					1.14	0.36–3.64
	Dominican × $15,000–$34,999			1.79	0.83–3.83					2.16 +	0.95–4.91
	Dominican × $35,000–$74,999			1.92	0.81–4.56					2.48 +	0.94–6.54
	Dominican × $75,000			0.38	0.07–2.00					0.39	0.09–1.77
	Dominican × Unknown			1.85	0.54–6.31					1.96	0.61–6.27
	Other/Multiple × $15,000–$34,999			0.79	0.31–2.00					0.97	0.28–3.41
	Other/Multiple × $35,000–$74,999			0.67	0.30–1.50					0.87	0.31–2.43
	Other/Multiple × $75,000+			1.77	0.49–6.33					2.30	0.49–10.88
	Other/Multiple × Unknown			1.62	0.52–5.06					2.19	0.60–7.93
Education (Less than HS Ref)											
	HS Graduate/GED	0.92 +	0.84–1.01			0.83 **	0.74–0.94			0.88 *	0.78–1.00
	Some College	0.99	0.90–1.09			0.91	0.81–1.03			0.94	0.83–1.06
	College Degree	0.90 *	0.82–0.98			0.70 ***	0.62–0.78			0.86 *	0.76–0.96
Race and Ethnicity × Education											
	Mexican × HS Graduate/GED					1.22	0.95–1.56			1.16	0.90–1.48
	Mexican × Some College					1.28 +	0.99–1.66			1.28 +	0.99–1.66
	Mexican × College Degree					1.40 **	1.10–1.78			1.28 *	1.00–1.63
	Central/South × HS Graduate/GED					0.98	0.66–1.47			0.92	0.61–1.38
	Central/South × Some College					1.00	0.68–1.47			0.98	0.66–1.44
	Central/South × College Degree					1.04	0.73–1.48			0.88	0.61–1.27
	Puerto Rican × HS Graduate/GED					1.10	0.63–1.93			1.12	0.66–1.93
	Puerto Rican × Some College					1.07	0.60–1.92			1.16	0.66–2.04
	Puerto Rican × College Degree					1.17	0.68–2.00			1.20	0.69–2.06
	Cuban × HS Graduate/GED					1.10	0.47–2.62			1.12	0.45–2.79
	Cuban × Some College					1.48	0.59–3.70			1.49	0.53–4.15
	Cuban × College Degree					1.46	0.61–3.51			1.19	0.45–3.19
	Dominican × HS Graduate/GE					0.68	0.32–1.47			0.64	0.27–1.50
	Dominican × Some College					0.68	0.28–1.68			0.69	0.27–1.75
	Dominican × College Degree					0.99	0.47–2.08			0.96	0.43–2.12
	Other/Multiple × HS Graduate/GED					0.57	0.15–2.15			0.66	0.18–2.49
	Other/Multiple × Some College					1.02	0.31–3.40			0.96	0.29–3.13
	Other/Multiple × College Degree					1.12	0.35–3.60			1.02	0.31–3.32
Employment Status (Employed Ref)		1.45 ***	1.36–1.56					1.69 ***	1.57–1.82	1.44 ***	1.34–1.56
Race and Ethnicity × Employment Status											
	Mexican × Unemployed							0.83	0.67–1.04	0.92	0.72–1.16
	Central/South × Unemployed							0.76	0.53–1.10	0.85	0.59–1.22
	Puerto Rican × Unemployed							1.25	0.79–1.98	1.35	0.85–2.16
	Cuban × Unemployed							2.24 +	0.86–5.78	2.37 +	0.86–6.57
	Dominican × Unemployed							2.14 +	0.97–4.72	2.93 *	1.17–7.34
	Other/Multiple × Unemployed							1.55	0.69–3.52	1.64	0.58–4.65
Observations		71,064		71,064		71,064		71,064		71,064	

Notes: *** *p* < 0.001, ** *p* < 0.01, * *p* < 0.05, + *p* < 0.1; Psychological distress (dependent variable) was coded with three categories (0 = none, 1 = moderate, 2 = severe); Ref = Referent; Latinx category names have been shortened in some cases for formatting purposes to Central/South = Central/South American, Mexican = Mexican/Mexican American, Other/Multiple = Other/Multiple Latinx; ⊥ = Referent for Poor/Fair Health includes Good, Very Good, and Excellent Health.

## Data Availability

The 2014–2018 National Health Interview Survey datasets used for the analyses in this study are available online from the National Center for Health Statistics (https://www.cdc.gov/nchs/nhis/index.htm, accessed on 2 October 2021).

## Supplementary Materials

All necessary materials and documentation, including questionnaires and survey methodology for the National Health Interview Survey, are available online from the National Center for Health Statistics (https://www.cdc.gov/nchs/nhis/index.htm, accessed on 2 October 2021).
